# Ocrelizumab effect on humoral and cellular immunity in multiple sclerosis and its clinical correlates: a 3-year observational study

**DOI:** 10.1007/s00415-022-11350-1

**Published:** 2022-09-01

**Authors:** Nicola Capasso, Raffaele Palladino, Vincenza Cerbone, Antonio Luca Spiezia, Bianca Covelli, Antonia Fiore, Roberta Lanzillo, Antonio Carotenuto, Maria Petracca, Lucia Stanziola, Giulia Scalia, Vincenzo Brescia Morra, Marcello Moccia

**Affiliations:** 1 Multiple Sclerosis Unit, Federico II University Hospital, Via Sergio Pansini 5, 80131 Naples, Italy; 2 Department of Public Health, Federico II University of Naples, Naples, Italy; 3 Centre for Advanced Biotechnology (CEINGE), Naples, Italy; 4 Department of Neuroscience, Reproductive Sciences and Odontostomatology, Federico II University of Naples, Naples, Italy; 5 Department of Translational Medical Sciences, Federico II University of Naples, Naples, Italy; 6 Department of Human Neuroscience, Sapienza University of Rome, Rome, Italy; 7 Department of Molecular Medicine and Medical Biotechnology, Federico II University of Naples, Via Sergio Pansini 5, 80131 Naples, Italy

**Keywords:** Multiple sclerosis, Ocrelizumab, Lymphocytes, Immunoglobulins

## Abstract

**Objective:**

We aim to evaluate 3-year effects of ocrelizumab (humanized anti-CD20 monoclonal antibody for the treatment of multiple sclerosis (MS)) on lymphocytes, neutrophils and immunoglobulins: (1) when compared with pre-infusion assessment; (2) over the course of treatment; and (3) possible clinical correlates of the observed immunological modifications.

**Methods:**

This real-world observational cohort study has been conducted on prospectively collected data from 78 MS patients (mean age 47.8 ± 10.5 years; females 48.7%) commencing on ocrelizumab from 2018, with mean follow-up of 36.5 ± 6.8 months. Clinical data and blood samples were collected every three months. Total lymphocyte count and subpopulations were assessed on peripheral blood using flow cytometry. Serum immunoglobulins were evaluated with nephelometry.

**Results:**

When compared with pre-infusion values, we observed reduction of total, CD19 and CD20 lymphocyte counts; however, after the first infusion, their levels remained substantially stable. Over time we observed a progressive reduction of CD8 lymphocytes, while no changes were observed for CD4, CD27, CD3CD27, and CD19CD27. After the first infusion, we observed reduction in IgG, which further decreased during the follow-up. Higher probability of EDSS progression was associated with reduced modulation of CD8 lymphocytes.

**Interpretation:**

Ocrelizumab affects both humoral and cellular immune responses. Disability progression over the follow-up was associated with lower CD8 cytotoxic T-lymphocyte reduction. Changes in humoral response are immediate and sustained, while modulation of cellular immunity occurs progressively through regular re-treatment, and is related to clinical stability.

## Introduction

Disease-modifying treatments (DMTs) for multiple sclerosis (MS) imply the chronic modulation and/or depletion of humoral and/or cellular components of immunity. In particular, a humanized anti-CD20 monoclonal antibody (ocrelizumab) is approved for relapsing–remitting MS (RRMS), active secondary progressive MS (SPMS) and primary progressive MS (PPMS), being effective on both relapses and disability progression, as also confirmed in real-world studies [1–3]. The effect of ocrelizumab is thought to be mediated by the depletion of B cells which plays a crucial role in the pathogenesis of MS by producing pro-inflammatory cytokines, interacting with other inflammatory cells (i.e., macrophages, natural killer cells, and cytotoxic T lymphocytes), and stimulating antibody production [4–8]. Further, B cells also act as antigen-presenting cells being involved in the activation of T cells in secondary lymphoid tissues, and thus contributing to chronic non-resolving inflammation [9, 10].

Over six months after the first infusion of ocrelizumab, a reduction of B and T cell subpopulations (CD19, CD20, CD4, CD8) is already seen [11, 12]. Using 7-year clinical trial data, Hauser and colleagues confirmed the action of ocrelizumab on both B and T cells, and also showed a reduction of serum IgM, IgG and IgA, when compared with pre-ocrelizumab levels [13]. However, authors only investigated changes between pre-ocrelizumab immunological status and follow-up, while any further changes occurring after the first infusion could have implications on risk of infection and on vaccine response in patients on chronic treatment with ocrelizumab [14–17]. Also, the effect of ocrelizumab on the activation of B and T cells (from naïve to active) remained unexplored.

In the present 3-years real-world observational cohort study, we aim to evaluate: (1) changes in total lymphocyte count, lymphocyte subpopulations (CD19 and CD20 B cells, CD4 T helper cells, CD8 cytotoxic T cells, CD27 activated T cells, CD3CD27 naïve and central memory T cells, CD19CD27 memory B cells), neutrophils and immunoglobulins (IgG, IgA and IgM) when compared with pre-infusion assessment; (2) any further changes after the first infusion; and (3) possible clinical correlates of the observed immunological changes.

## Methods

### Study design and population

This real-world observational cohort study was conducted at the MS Unit of the Federico II University Hospital of Naples, Italy, on prospectively collected data from 2018 to 2021. The study was approved by the Federico II Ethics Committee (355/19). All patients signed informed consent authorizing the use of anonymized data in line with data protection regulation (GDPR EU2016/679). The present study was performed in accordance with good clinical practice and Declaration of Helsinki.

Inclusion criteria were: (1) patients with MS at first treatment with ocrelizumab from 2018 to 2021; (2) continued ocrelizumab treatment for at least 2 years (with < 4 weeks of flexibility in 6-month infusion intervals) [18, 19]; (3) availability of clinical and laboratory data at baseline (before first ocrelizumab infusion) and over at least 2 years. Exclusion criteria were: (1) age < 18 years; (2) pregnancy; (3) concomitant diseases (i.e., immunodeficiency diseases) or treatments (i.e., chemotherapy, immunosuppressive therapy) affecting the immune system.

First, ocrelizumab infusion was split into two 300 mg infusions (300 mg at week day 0 and 14). The date of the first infusion was counted as baseline. Following infusions were performed every 6 months, with < 4 weeks of flexibility to 6-month infusion intervals, which is deemed to be within regular infusion dosing [18, 19]. Clinical evaluations and blood sample collection were performed at least every three months over the follow-up period.

### Laboratory variables

**Fig. 1:**
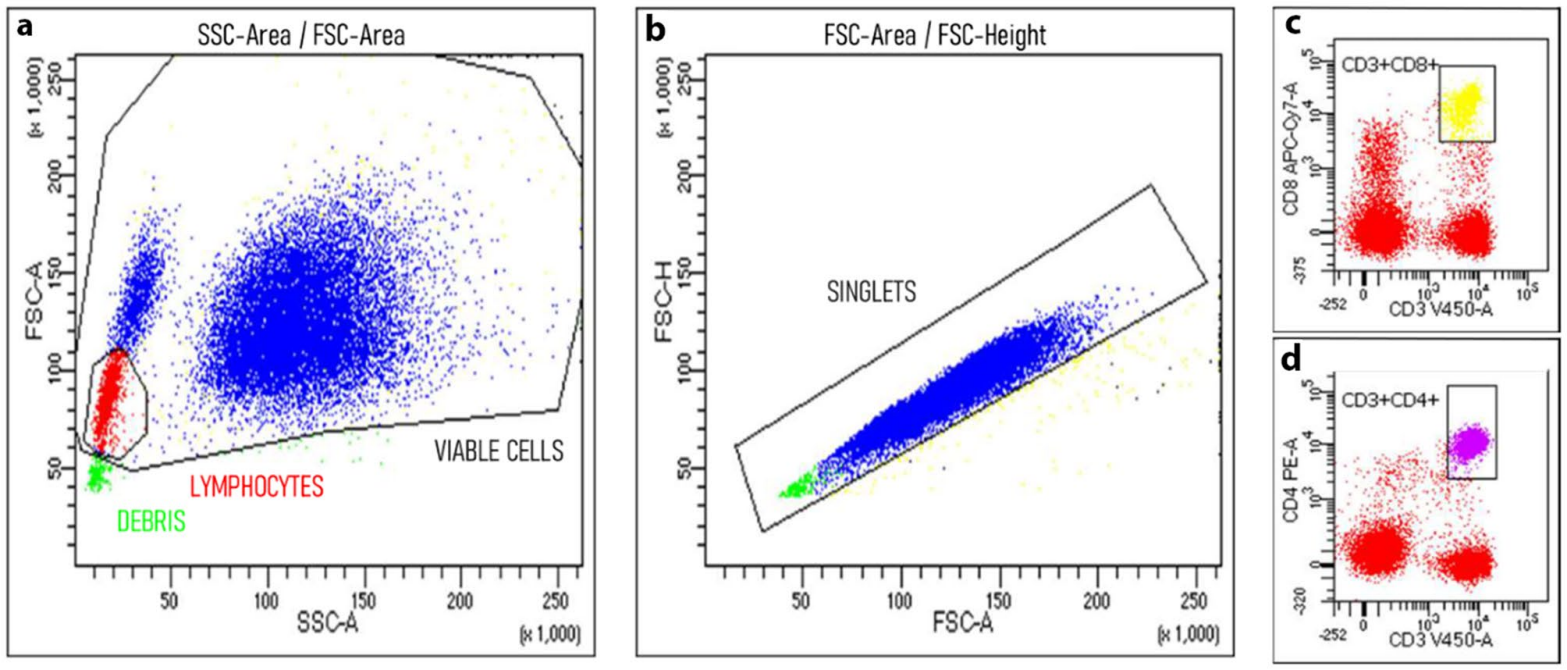
Flow cytometry gating strategy. The dot plots show the subsequent gates that were drawn to analyze the lymphocytes. SSC-Area and FSC-Area were plotted to exclude debris and to identify viable cells, among which the lymphocyte region was defined (**a**). Then, FSC-Area and FSC-Height were used to exclude doublets (**b**). From this region of lymphocyte singlets, plots were constructed to define lymphocyte subpopulations based on CD expression (e.g., CD3 + CD4 + in **c**, CD3 + CD8 + in **d**)

An aliquot (50 μL) of anti-coagulated ethylene-diamine-tetra-acetic acid (EDTA) whole fresh blood (within 12 h) was incubated at 4 °C for 30 min in the presence of appropriate amounts of monoclonal antibodies. The mixtures were then diluted 1:20 in ammonium chloride lysing solution, incubated at room temperature for 10 min and finally washed prior to flow cytometric analysis with the FACSCanto II flow cytometer (Becton Dickinson, San Jose, CA, USA). Samples were analyzed on FACSDiva software (BD Bioscience, San Jose, CA, USA). The following antigens were analyzed: CD4 PE (from BD San Diego, CA, USA), CD8 APCcy7 (from Beckman Coulter, Marseille Cedex 9, France), CD20 FITC (from BD San Diego, CA, USA), CD19 APC (from Beckman Coulter, Marseille Cedex 9, France), CD45 PerCP (from BD San Diego, CA, USA), CD27 HV500 (from BD San Diego, CA, USA), CD3 Pacific Blu (from Beckman Coulter, Marseille Cedex 9, France). B and T lymphocytes were gated on forward scatter (FSC) and side scatter (SSC) parameters, identifying 50,000 events. Gating strategy is shown in Fig. 1. The lowest level of detection was 10^–4^ (as such, zero corresponds to a level below 1/10,000 cells). For lymphocyte absolute count, we coupled cytometry to complete blood count on hematological counter (double platform). Laboratory procedures were performed in accordance with UK-NEQAS quality standards (https://ukneqas.org.uk/). For quantitative testing of serum immunoglobulins, we used nephelometry with a wavelength of 840 nm (BN™ II System, Siemens Healthcare, Erlangen, Germany), in accordance with manufacturer instructions. Reference curves were generated by multi-point calibration. Serum samples were automatically diluted 1:400 (IgG), 1:20 (IgA) or 1:5 in the low concentration assay (IgAs and IgMs). Reference values were derived from Italian population [20].

### Demographic and clinical variables

At baseline, age, sex, disease duration (time from symptom onset to baseline visit), disease subtype (RRMS, SPMS, PPMS), and previous disease-modifying treatments (DMTs) were collected as per clinical practice. Disability was scored with the Expanded Disability Status Scale (EDSS), by certified examiners. EDSS progression at last available follow-up was defined as increase in EDSS by 1 point if baseline EDSS was 5.5 or lower, or increase in EDSS by 0.5 point if baseline EDSS was above 5.5 [21]. Relapses were recorded on the occasion of clinical consultations and infusions. Side effects were also collected on the occasion of clinical consultations and infusions; however, considering the retrospective nature of the study, we only referred to serious adverse events, which are less likely to be missed in clinical practice (defined as reaction that results in death, is life-threatening, requires hospitalization or prolongation of existing hospitalization, results in persistent or significant disability or incapacity, or is a birth defect).

### Power calculation

Considering a normal distribution of variables to be analyzed in regression models, and a 30% effect size [13], a sample of 78 patients would be able to achieve 87% power with 5% α error.

### Statistics

Study variables are presented as mean (standard deviation), median (range), or number (percent) as appropriate.

Changes in laboratory variables were explored using mixed-effect regression models including each laboratory variable, in turn, as dependent variable, and infusion as independent variable; covariates were age, sex, previous DMT, and follow-up duration. Patients were included in the models as random intercept to account for the hierarchical structure of the data. As unit of analysis was the infusion, in case of multiple outcome records corresponding to the same infusion, the statistical model averaged them. Our statistical references were laboratory variables at baseline (first infusion), and, then, after first infusion, to assess changes over time when compared with before ocrelizumab treatment and following its first infusion, respectively. Clinical correlates of immunological modifications (i.e., relapse occurrence, side effects and EDSS progression) were explored using logistic regression models including each clinical variable (EDSS progression, relapse occurrence, serious adverse events), in turn, as dependent variable, and each laboratory variable, in turn, as independent variable; covariates were age, sex, previous DMT, and follow-up duration.

Results are presented as coefficients (Coeff), odds ratio (OR), 95% confidence intervals (95%CI), and p-values. Statistical analyses were performed using Stata 17.0.

## Results

We included 78 MS patients (age 47.8 ± 10.5 years; sex 48.7% females), with 36.5 ± 6.8 months of follow-up. 51.3% patients had 7 infusions of ocrelizumab (ranging from 5 to 8 infusions). Demographic, clinical and treatment features are reported in Table 1.

**Table 1:** Demographic, clinical and treatment features

	*N* = *78*
*Baseline*	
Age, years	47.8 ± 10.5
Sex, females (%)	38 (48.7%)
Disease duration, years	13.2 ± 8.6
EDSS at baseline, median (range)	3.5 (1.5–7.0)
*Disease subtype*	
RR	38 (48.7%)
SP	9 (11.5%)
PP	31 (39.8%)
*Previous DMT*	
Alemtuzumab	2 (2.6%)
Dimethyl fumarate	13 (16.7%)
Fingolimod	15 (19.2%)
Glatiramer acetate	8 (10.2%)
Interferon beta	5 (6.4%)
Natalizumab	6 (7.7%)
Teriflunomide	9 (11.6%)
Treatment naïve	20 (25.6%)
*Follow-up*	
Follow-up duration, months	36.5 ± 6.8
*Number of ocrelizumab infusions*	
5	14 (17.9%)
6	6 (7.7%)
7	40 (51.3%)
8	18 (23.1%)
EDSS at follow-up, median (range)	4.0 (1.5–8.0)
EDSS progression, *n* (%)	26 (33.3%)
Relapses, *n* (%)	2 (2.6%)
*Serious adverse events*	
Infections	4 (5.1%)
Malignancy	4 (5.1%)

**Fig. 2:**
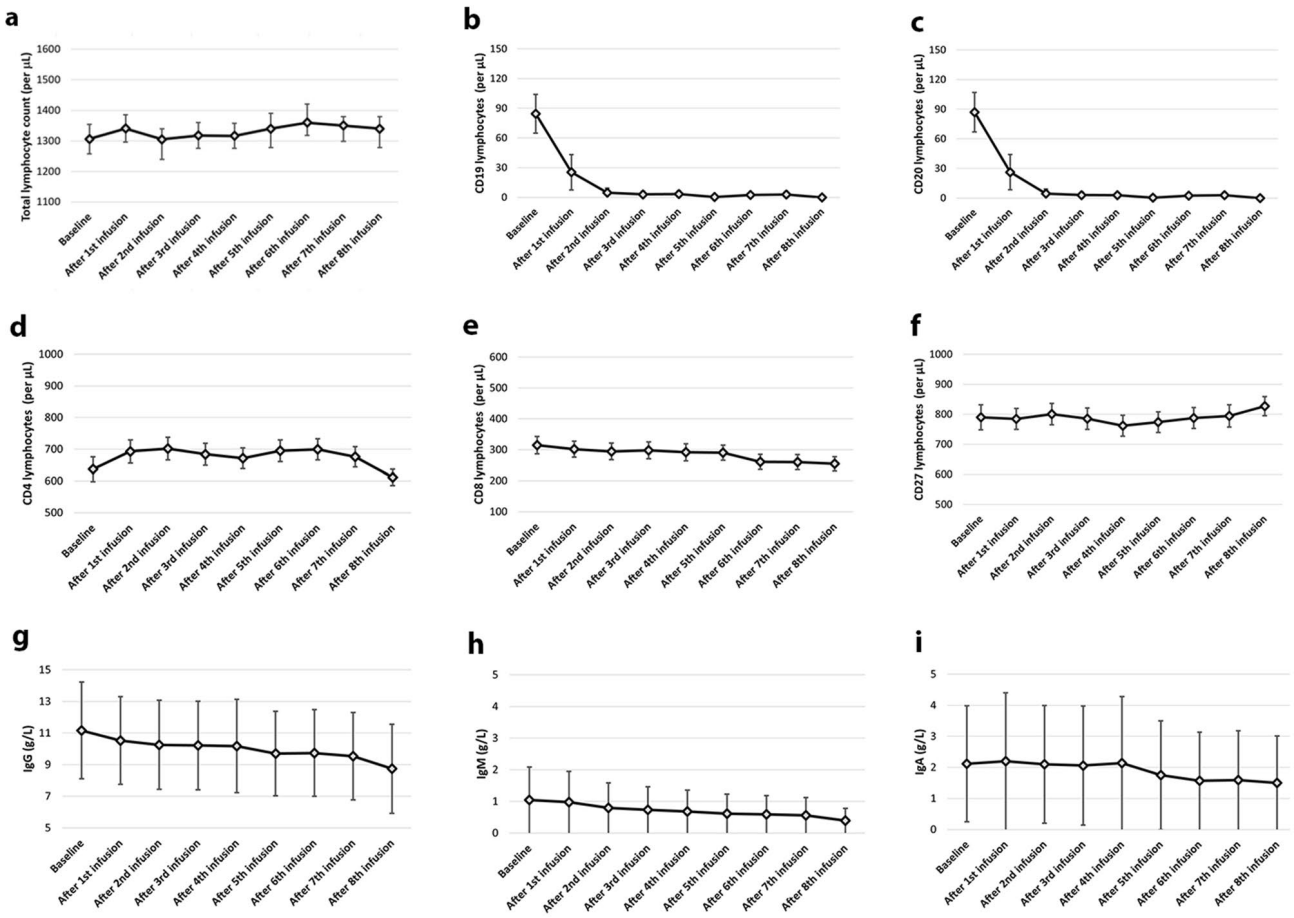
Laboratory changes over time. Profile plots show changes in total lymphocyte count (**a**), CD19 lymphocytes (**b**), CD20 lymphocytes (**c**), CD4 lymphocytes (**d**), CD8 lymphocytes (**e**), CD27 lymphocytes (**f**), IgG (**g**), IgM (**h**) and IgA (**i**)

When compared with pre-infusion values, white blood cell count initially decreased after 2nd to 4th infusion, but then increased after 6th to 8th infusion; total lymphocyte count decreased after 1st and 2nd infusion; CD19 and CD20 lymphocytes decreased across all infusions; CD8 lymphocytes decreased after 3rd to 8th infusion; CD4/CD8 ratio increased after 2nd to 8th infusion; and IgG decreased after 1st to 8th infusion. No changes were observed for neutrophils, CD4 lymphocytes, CD27 lymphocytes, CD3CD27 lymphocytes, CD19CD27 lymphocytes, IgM and IgA (Table 2; Fig. 2).

**Table 2:** Laboratory variables over time

		Baseline	After 1st infusion	After 2nd infusion	After 3rd infusion	After 4th infusion	After 5th infusion	After 6th infusion	After 7th infusion	After 8th infusion
WBC	Coeff	Reference	− 264.09	**− 703.40**	**− 1147.99**	**− 1624.24**	− 921.50	**2080.78**	**2332.22**	**5704.85**
4500 to 11,000 cells per μL	95% CI		− 773.24	**− 1337.32**	**− 1976.85**	**− 2688.40**	− 2245.07	**539.68**	**511.54**	**3310.19**
			245.05	**− 69.48**	**− 319.12**	**− 560.07**	402.06	**3621.89**	**4152.90**	**8099.51**
	*P* value		0.30	**0.03**	** < 0.01**	** < 0.01**	0.17	** < 0.01**	**0.01**	** < 0.01**
	Coeff		Reference	**− 498.41**	**− 986.68**	**− 1526.80**	**− 874.46**	**2071.16**	**2273.03**	**5605.35**
	95% CI			**− 906.79**	**− 1620.93**	**− 2423.15**	**− 2054.42**	**661.41**	**564.87**	**3281.48**
				**− 90.04**	**− 352.42**	**− 630.44**	**305.48**	**3480.91**	**3981.19**	**7929.22**
	*P* value			**0.01**	** < 0.01**	** < 0.01**	**0.14**	** < 0.01**	** < 0.01**	** < 0.01**
Neutrophils	Coeff	Reference	− 102.93	103.28	381.71	775.87	782.65	767.52	836.67	1804.06
1800 to 7000 cells per μL	95% CI		− 485.34	− 394.58	− 289.27	− 103.06	− 317.39	− 524.46	− 699.48	− 154.33
			279.47	601.15	1052.71	1654.81	1882.71	2059.51	2372.83	3762.46
	*P* value		0.59	0.68	0.26	0.08	0.16	0.24	0.28	0.07
	Coeff		Reference	243.01	**540.65**	**962.34**	**1002.74**	999.66	114.35	**2067.16**
	95% CI			− 68.18	**37.21**	**237.48**	**48.37**	− 151.08	− 285.97	**235.20**
				554.20	**1044.09**	**1687.20**	**1957.11**	2150.42	2514.68	**3899.11**
	*P* value			0.12	**0.03**	** < 0.01**	**0.04**	0.08	0.11	**0.02**
Lymphocytes	Coeff	Reference	**− 104.98**	**− 137.23**	− 131.05	− 131.24	− 106.61	− 45.14	39.45	258.79
1000 to 4800 cells per μL	95% CI		**− 192.26**	**− 252.21**	− 287.22	− 336.86	− 364.30	− 348.47	− 321.79	− 198.39
			**− 17.70**	**− 22.24**	25.11	74.36	151.08	258.19	400.69	715.97
	*P* value		**0.01**	**0.01**	0.10	0.21	0.41	0.77	0.83	0.26
	Coeff		Reference	− 47.74	− 52.33	− 68.44	− 55.95	− 7.07	− 65.28	281.11
	95% CI			− 119.75	− 169.86	− 238.35	− 279.68	− 277.37	− 263.78	− 145.50
				24.25	65.20	101.47	167.77	263.23	394.34	707.72
	*P* value			0.19	0.38	0.43	0.62	0.95	0.69	0.19
CD19	Coeff	Reference	**− 59.18**	**− 77.78**	**− 77.69**	**− 75.63**	**− 76.04**	**− 72.77**	**− 71.26**	**− 69.00**
73 to 654 cells per μL	95% CI		**− 76.34**	**− 98.42**	**− 104.12**	**− 109.12**	**− 117.44**	**− 121.08**	**− 128.19**	**− 142.88**
			**− 42.03**	**− 57.14**	**− 51.26**	**− 42.14**	**− 34.64**	**− 24.46**	**− 14.33**	**− 4.87**
	*P* value		** < 0.01**	** < 0.01**	** < 0.01**	** < 0.01**	** < 0.01**	** < 0.01**	**0.01**	**0.03**
	Coeff		Reference	**− 17.37**	− 16.30	− 13.22	− 12.51	− 8.04	− 5.94	− 3.50
	95% CI			**− 29.01**	− 34.18	− 38.35	− 45.54	− 47.78	− 54.07	− 68.20
				**− 5.74**	1.56	11.91	20.52	31.70	42.18	61.20
	*P* value			** < 0.01**	0.07	0.30	0.45	0.69	0.80	0.91
CD20	Coeff	Reference	**− 59.90**	**− 78.06**	**− 76.37**	**− 73.22**	**− 71.69**	**− 66.92**	**− 64.48**	**− 61.76**
73 to 654 cells per μL	95% CI		**− 77.47**	**− 99.40**	**− 103.74**	**− 107.88**	**− 114.53**	**− 116.85**	**− 123.31**	**− 137.80**
			**− 42.33**	**− 56.73**	**− 49.01**	**− 38.56**	**− 28.85**	**− 16.99**	**− 5.65**	**− 14.27**
	*P* value		** < 0.01**	** < 0.01**	** < 0.01**	** < 0.01**	** < 0.01**	** < 0.01**	**0.03**	**0.02**
	Coeff		Reference	**− 18.47**	− 17.40	− 14.79	− 13.80	− 9.34	− 7.52	− 5.44
	95% CI			**− 30.30**	− 35.56	− 40.31	− 47.38	− 49.70	− 56.44	− 71.21
				**− 6.65**	0.76	10.73	19.77	31.01	41.39	60.32
	*P* value			** < 0.01**	0.06	0.25	0.42	0.65	0.76	0.87
CD4	Coeff	Reference	− 26.18	− 31.48	− 27.56	− 26.87	− 22.33	− 34.34	− 40.20	− 43.18
493 to 1666 cells per μL	95% CI		− 80.66	− 103.92	− 126.55	− 157.48	− 185.81	− 227.71	− 271.29	− 274.42
			28.29	40.94	71.42	103.73	141.14	159.01	190.88	302.73
	*P* value		0.34	0.39	0.58	0.68	0.78	0.72	0.73	0.92
	Coeff		Reference	− 12.88	− 13.03	− 19.28	− 21.44	− 39.16	− 51.50	− 53.72
	95% CI			− 58.39	− 87.84	− 127.60	− 163.78	− 212.03	− 262.84	− 265.17
				32.61	61.77	89.04	120.89	133.70	159.82	157.71
	*P* value			0.57	0.73	0.72	0.76	0.65	0.63	0.61
CD8	Coeff	Reference	− 5.02	− 26.74	**− 47.43**	**− 49.76**	**− 73.49**	**− 77.35**	**− 86.33**	**− 228.89**
224 to 1112 cells per μL	95% CI		− 31.75	− 54.21	**− 76.08**	**− 79.83**	**− 105.33**	**− 180.72**	**− 142.67**	**− 426.50**
			21.71	0.71	**− 18.78**	**− 19.70**	**− 41.65**	**− 26.00**	**− 29.98**	**− 31.28**
	*P* value		0.71	0.05	** < 0.01**	** < 0.01**	** < 0.01**	**0.01**	** < 0.01**	**0.02**
	Coeff		Reference	− 24.23	**− 61.53**	**− 74.66**	− 83.45	− 59.47	− 51.36	− 33.92
	95% CI			− 53.25	**− 109.16**	**− 143.57**	− 173.99	− 169.40	− 185.73	− 205.77
				4.78	**− 13.90**	**− 5.74**	7.08	50.45	83.00	137.92
	*P* value			0.10	**0.01**	**0.03**	0.07	0.28	0.45	0.69
CD4/CD8 ratio	Coeff	Reference	0.26	**0.34**	**0.71**	**0.73**	**0.99**	**0.96**	**0.82**	**0.93**
	95% CI		− 0.01	**0.06**	**0.41**	**0.42**	**0.67**	**0.76**	**0.14**	**0.11**
			0.53	**0.62**	**1.00**	**1.04**	**1.32**	**1.64**	**1.50**	**2.98**
	*P* value		0.06	**0.01**	** < 0.01**	** < 0.01**	** < 0.01**	**0.01**	**0.01**	**0.01**
	Coeff		Reference	− 0.12	0.04	− 0.14	− 0.07	− 0.05	− 0.56	− 0.62
	95% CI			− 0.45	− 0.49	− 0.93	− 1.12	− 1.34	− 2.16	− 3.28
				0.19	0.59	0.65	0.96	1.24	1.02	2.03
	*P* value			0.44	0.86	0.72	0.88	0.93	0.48	0.64
CD27	Coeff	Reference	54.95	99.20	97.23	105.13	118.05	115.15	169.87	90.83
	95% CI		− 97.24	− 57.50	− 73.95	− 90.08	− 105.86	− 138.82	− 122.47	− 256.37
			207.15	255.92	268.41	300.36	341.97	369.12	462.21	438.03
	*P* value		0.47	0.21	0.26	0.29	0.30	0.37	0.25	0.60
	Coeff		Reference	38.58	32.41	26.43	46.12	41.32	94.08	158.84
	95% CI			− 24.83	− 58.98	− 92.21	− 121.74	− 163.22	− 155.44	− 62.86
				102.01	123.81	165.09	213.99	245.88	343.61	380.55
	*P* value			0.23	0.48	0.57	0.59	0.69	0.46	0.16
CD3CD27	Coeff	Reference	26.78	30.37	69.12	72.42	61.04	74.07	67.31	100.32
	95% CI		− 124.74	− 147.88	− 110.51	− 120.91	− 156.26	− 172.67	− 211.02	− 218.49
			178.31	208.64	248.89	265.75	278.35	320.81	345.65	419.14
	*P* value		0.72	0.73	0.45	0.46	0.58	0.55	0.63	0.53
	Coeff		Reference	30.37	32.96	20.43	31.27	24.79	56.24	32.68
	95% CI			− 48.91	− 71.23	− 120.32	− 150.43	− 196.21	− 212.68	− 119.49
				109.66	137.15	161.20	212.98	245.80	325.17	184.87
	*P* value			0.45	0.53	0.77	0.73	0.82	0.68	0.67
CD19CD27	Coeff	Reference	− 5.20	1.96	8.88	29.35	24.86	31.91	38.57	44.29
	95% CI		− 51.30	− 43.99	− 39.70	− 23.89	− 34.67	− 33.69	− 34.47	− 41.58
			40.89	47.93	57.47	82.61	84.40	97.52	111.62	130.18
	*P* value		0.82	0.93	0.72	0.28	0.41	0.34	0.30	0.31
	Coeff		Reference	7.16	14.08	34.57	30.11	37.16	43.80	49.55
	95% CI			− 13.42	− 10.85	2.25	− 11.13	− 12.00	− 14.70	− 24.46
				27.75	39.02	66.88	71.35	86.33	102.31	123.57
	*P* value			0.49	0.26	0.03	0.15	0.13	0.14	0.18
IgG	Coeff	Reference	**− 0.70**	**− 1.01**	**− 0.97**	**− 1.21**	**− 1.66**	**− 1.94**	**− 2.52**	**− 3.01**
7.37 to 16.07 g/L	95% CI		**− 1.11**	**− 1.53**	**− 1.67**	**− 2.14**	**− 2.81**	**− 3.29**	**− 4.15**	**− 5.01**
			**− 0.29**	**− 0.48**	**− 0.26**	**− 0.29**	**− 0.51**	**− 0.59**	**− 0.90**	**− 1.01**
	*P* value		** < 0.01**	** < 0.01**	** < 0.01**	**0.01**	** < 0.01**	** < 0.01**	** < 0.01**	** < 0.01**
	Coeff		Reference	**− 0.35**	− 0.35	− 0.65	**− 1.15**	**− 1.48**	**− 2.08**	**− 2.61**
	95% CI			**− 0.65**	− 0.85	− 1.38	**− 2.11**	**− 2.63**	**− 3.51**	**− 4.41**
				**− 0.05**	0.14	0.07	**− 0.19**	**− 0.32**	**− 0.64**	**− 0.81**
	*P* value			**0.02**	0.16	0.07	**0.01**	**0.01**	** < 0.01**	** < 0.01**
IgM	Coeff	Reference	− 0.08	− 0.13	− 0.09	− 0.01	0.04	0.20	0.07	0.21
0.40 to 2.30 g/L	95% CI		− 0.23	− 0.32	− 0.33	− 0.34	− 0.35	− 0.26	− 0.49	− 0.48
			0.06	0.04	0.15	0.30	0.45	0.68	0.64	0.91
	*P* value		0.25	0.14	0.47	0.90	0.82	0.38	0.80	0.54
	Coeff		Reference	− 0.05	− 0.01	0.05	0.11	0.27	0.13	0.27
	95% CI			− 0.16	− 0.19	− 0.21	− 0.23	− 0.15	− 0.39	− 0.38
				0.05	0.17	0.32	0.46	0.69	0.65	0.93
	*P* value			0.31	0.89	0.70	0.52	0.21	0.62	0.41
IgA	Coeff	Reference	0.21	0.05	− 0.02	− 0.02	− 0.33	− 0.43	0.51	− 0.47
0.70 to 4.00 g/L	95% CI		− 0.24	− 0.52	− 0.78	− 1.02	− 1.56	− 1.88	− 2.25	− 2.62
			0.66	0.62	0.73	0.96	0.90	1.00	1.21	1.67
	*P* value		0.35	0.85	0.95	0.95	0.59	0.55	0.56	0.66
	Coeff		Reference	− 0.15	− 0.23	− 0.23	− 0.53	− 0.63	− 0.71	− 0.66
	95% CI			− 0.50	− 0.80	− 1.06	− 1.62	− 1.95	− 2.34	− 2.73
				0.19	0.34	0.60	0.55	0.68	0.91	1.40
	*P* value			0.38	0.43	0.58	0.33	0.34	0.39	0.52

Table shows coefficients (Coeff), 95% confidence intervals (95% CI), and *p* values from mixed-effect regression models including laboratory variables, in turn, as dependent variable, and infusion number as independent variable; covariates were age, sex, previous DMT, and follow-up duration. First, our statistical references were laboratory variables at baseline (first infusion), and, then, after 1st infusion, to assess changes over time when compared with before ocrelizumab treatment and following its first infusion, respectively. Significant results (*p* < 0.05) are reported in bold. Normal ranges are reported, as available

When compared with values after 1st infusion, white blood cell count initially decreased after 2nd to 5th infusion, but then increased after 6th to 8th infusion; neutrophils increased after 3rd to 8th infusion; CD19 and CD20 lymphocytes decreased after 2nd infusion; CD8 lymphocytes decreased after 3rd to 4th infusion; and IgG decreased after 2nd to 8th infusion. No changes were observed for total lymphocyte count, CD4 lymphocytes, CD4/CD8 ratio, CD27 lymphocytes, CD3CD27 lymphocytes, CD19CD27 lymphocytes, IgM and IgA (Table 2).

Over 36 months of follow-up, 26 patients (33.3%) had EDSS progression. The probability of EDSS progression was associated with higher CD8 (OR = 1.01; 95 CI = 1.00, 1.05; *p* = 0.02), and lower CD4/CD8 ratio (OR = 0.67; 95 CI = 0.46, 0.96; *p* = 0.03); no significant associations were found for WBC, neutrophils, total lymphocytes and other subsets (CD19, CD20, CD4, CD27, CD3CD27, and CD19CD27) (Table 3). We recorded two relapses, and eight cases of serious adverse events in seven patients; in particular, serious adverse events were infections (2 pulmonary infections, 1 herpes zoster infection, and 1 urinary tract infection), and malignancies (2 skin cancer, 1 bladder cancer, and 1 leukemia). Statistical models for relapses and side effects, separately, failed due to multicollinearity.

**Table 3:** EDSS progression and laboratory variables

	OR	95% CI		*p* value
WBC	1.00	0.99	1.01	0.91
Neutrophils	0.99	0.98	1.01	0.58
Lymphocytes	1.00	0.99	1.01	0.76
CD19	0.98	0.95	1.02	0.42
CD20	0.99	0.99	1.01	0.49
CD4	0.99	0.97	1.01	0.19
CD8	**1.01**	**1.00**	**1.05**	**0.02**
CD27	0.99	0.99	1.00	0.35
CD3CD27	0.99	0.99	1.00	0.21
CD19CD27	0.99	0.99	1.01	0.96
CD4/CD8 ratio	**0.67**	**0.46**	**0.96**	**0.03**

Table shows odds ratio (OR), 95% confidence intervals (95% CI), and p values from logistic regression models including EDSS progression as dependent variable, and laboratory variables, in turn, as independent variables; covariates were age, sex, previous DMT, and follow-up duration. Significant results (*p* < 0.05) are reported in bold

## Discussion

Our study confirmed that ocrelizumab has wide range effects on both humoral and cellular immune response from its first infusion, and showed that most changes occurred from the first to the fourth infusions, thus pointing toward relative stability of the immunological profile afterward. Of note, cellular, but not humoral, immune effects of ocrelizumab were associated with disability progression, and occurred throughout the follow-up, suggesting that continuous treatment with ocrelizumab is needed to modulate the neurodegenerative aspects of MS.

In our study, we confirmed previous clinical trial extension results on white blood cells, which progressively decreased following the first infusion, but then came back to previous levels after year 2, and on neutrophils, which remained stable throughout the study, in the absence of cases of late-onset neutropenia [13, 22, 23]. Looking at B lymphocytes, CD19 and CD20 B lymphocytes were the first subpopulation hit by ocrelizumab, decreasing immediately after the first infusion and then remaining substantially stable. B lymphocytes play an important role in MS pathology in the context of both relapsing and progressive MS [24–26], promoting injury through direct activation of lymphocytes (CD8 cytotoxic T lymphocytes) [26–29], and other neuro-inflammatory cells (e.g., macrophages, microglia, astrocytes) [30, 31] or the production of pro-inflammatory cytokines [32]. In the central nervous system of MS patients, B cells are primarily located in the meninges and in the perivascular spaces, where they contribute to chronic aberrant compartmentalized inflammation [28, 33]. In animal models, treatment with anti-CD20 monoclonal antibodies eliminates CD20 B lymphocytes and disrupts B cell aggregates [34].

Antibody production is strictly dependent on B-mediated humoral response, and can directly contribute to MS inflammatory changes [35–37]. Accordingly, our results showed a reduction of IgG from the first infusion, alongside B lymphocytes, with progressive decrease over infusions, as already shown by clinical trial extension [13]. While the low levels of IgG have raised concerns on infectious risk and vaccine response in MS [15–17, 38], our study showed stable IgM levels and CD19CD27 B lymphocytes. In particular, CD19CD27 B lymphocytes in the peripheral blood are active B cells, but share the same antigens with plasma cells, located within lymphoid tissues and hereby producing immunoglobulins [39]. Taken together, these observations indirectly suggest that, while CD20 B lymphocytes are depleted, plasma cells remain unaffected, with normal possibility to mount humoral immune response against new antigens (IgM) [25], as preliminary shown in some cases [38].

Looking at T lymphocyte subpopulations, CD4 T lymphocytes remained substantially stable as previously showed in our feeder study [12], and also in clinical trial extension results [17]. On the contrary, CD8 T lymphocytes progressively decreased over infusions, as shown by regression coefficients and also confirmed by a concomitant drop of the CD4/CD8 ratio. However, we did not observe changes in CD27 activated T lymphocytes and CD3CD27 naïve cells (CD45RO−) and central memory T lymphocytes (CD45RO +), suggesting that ocrelizumab leaves unaffected the possibility to mount a cellular immune response while specifically targeting the aberrant immune response mediated by CD8 cytotoxic T lymphocytes. In keep with this, we found an association between increased probability of EDSS progression and reduced ocrelizumab-mediated modulation (i.e., higher levels) of CD8 T lymphocytes. While we need to acknowledge small effect size in this statistical model, this association is underlined by strong biological plausibility. Indeed, CD8 T lymphocytes directly contribute to non-resolving inflammation within the central nervous system, ultimately leading to demyelination and axon loss in both relapsing and progressive MS [30]. In particular, in progressive MS, inflammatory activity and demyelination is associated with infiltrates of tissue-resident CD8 cytotoxic T cells [40]. As such, the effect of ocrelizumab does not only depend on depletion of B lymphocytes, which is early event and substantially stable after the second infusion (using 6-months interval dosing), but also of CD8 cytotoxic T lymphocytes, which, on the contrary, occurs progressively during the course of treatments. Thus, while extending ocrelizumab dosing interval following B lymphocyte counts could be effective on the inflammatory aspects of MS (i.e., relapses, MRI lesions) [19], CD8 cytotoxic T lymphocytes progressively decrease during treatment and would very likely require regular infusions to be constantly modulated, with subsequent effect on disability progression [18]. In line with this, in a murine model, single administration of anti-CD20 monoclonal antibody was immediately effective on B cell aggregates in the absence of changes in other inflammatory cells [41].

Unfortunately, we were unable to provide more thoughtful insights on other clinical variables. We recorded relapses, which were too few to be analyzed statistically and correlated to laboratory variables. The low rate of relapses could be due to the strong and immediate anti-inflammatory effect of ocrelizumab [42], but also to the inclusion of progressive patients, representing about 50% of the population. We only had eight cases of serious adverse events (four infections, and four malignancies), whom statistical models failed due to the small number of observations. This could be due to the inclusion of serious adverse events only, the retrospective nature of the study, and/or the implementation of a program for infection screening and prophylaxis, which proved effective in reducing infections in patients receiving anti-CD20 agents for MS [43].

Additional limitations include the lack of more detailed clinical (e.g., walking and hand dexterity tests), and MRI (e.g., lesion load, atrophy) outcome measures, which would have been helpful for improved population characterization and treatment effect estimation [44, 45], but unfortunately not routinely collected across the follow-up. Finally, we did not include CD45RO in the panel, and thus were unable to differentiate CD3 + CD27 + cells into naïve and central memory T lymphocytes.

In conclusion, we provided real-world evidence on the mechanisms of action of ocrelizumab, and confirmed its effect on both cellular and humoral immunity. While the effect on B lymphocytes and, subsequently, on immunoglobulins is well known and expected, the mechanisms by which ocrelizumab reduces CD8 cytotoxic T lymphocytes deserves to be further explored also in light of its association with disability progression.

## References

[1] Ellwardt E, Rolfes L, Klein J (2020). Ocrelizumab initiation in patients with MS: a multicenter observational study. Neurol Neuroimmunol Neuroinflamm.

[2] Montalban X, Hauser SL, Kappos L (2017). Ocrelizumab versus placebo in primary progressive multiple sclerosis. N Engl J Med.

[3] Hauser SL, Bar-Or A, Comi G (2016). Ocrelizumab versus interferon β-1a in relapsing multiple sclerosis. N Engl J Med.

[4] Archelos JJ, Storch MK, Hartung MH-P (2000). The role of B cells and autoantibodies in multiple sclerosis. Ann Neurol.

[5] Bar-Or A, Fawaz L, Fan B (2010). Abnormal B-cell cytokine responses a trigger of T-cell-mediated disease in MS?. Ann Neurol.

[6] Anderson DR, Grillo-Löpez A, Varns C (1997). Targeted anti-cancer therapy using rituximab, a chimaeric anti-CD20 antibody (IDEC-C2B8) in the treatment of non-Hodgkin’s B-cell lymphoma. Biochem Soc Trans.

[7] Clynes RA, Towers TL, Presta LG, Ravetch JV (2000). Inhibitory Fc receptors modulate in vivo cytoxicity against tumor targets. Nat Med.

[8] Disanto G, Morahan JM, Barnett MH (2012). The evidence for a role of B cells in multiple sclerosis. Neurology.

[9] Christensen JR, Börnsen L, Ratzer R (2013). Systemic inflammation in progressive multiple sclerosis involves follicular T-Helper, Th17- and activated B-cells and correlates with progression. PLoS One.

[10] Tangye SG, Ma CS, Brink R, Deenick EK (2013). The good, the bad and the ugly-T FH cells in human health and disease. Nat Rev Immunol.

[11] Fernández-Velasco JI, Kuhle J, Monreal E (2021). Effect of ocrelizumab in blood leukocytes of patients with primary progressive MS. Neurol Neuroimmunol Neuroinflamm.

[12] Capasso N, Nozzolillo A, Scalia G (2021). Ocrelizumab depletes T-lymphocytes more than rituximab in multiple sclerosis. Mult Scler Relat Disord.

[13] Hauser SL, Kappos L, Montalban X (2021). Safety of ocrelizumab in patients with relapsing and primary progressive multiple sclerosis. Neurology.

[14] Zabalza A, Arrambide G, Tagliani P (2022). Humoral and cellular responses to SARS-CoV-2 in convalescent COVID-19 patients with multiple sclerosis. Neurol Neuroimmunol Neuroinflamm.

[15] Baker D, MacDougall A, Kang AS, Schmierer K, Gavin Giovannoni RD (2021). Seroconversion following COVID-19 vaccination: Can we optimize protective response in CD20-treated individuals?. Clin Exp Immunol.

[16] Tortorella C, Aiello A, Gasperini C (2022). Humoral- and T-cell–specific immune responses to SARS-CoV-2 mRNA vaccination in patients with MS using different disease-modifying therapies. Neurology.

[17] Baker D, MacDougall A, Kang AS (2022). CD19 B cell repopulation after ocrelizumab, alemtuzumab and cladribine: implications for SARS-CoV-2 vaccinations in multiple sclerosis. Mult Scler Relat Disord.

[18] van Lierop ZYGJ, Toorop AA, van Ballegoij WJC (2021). Personalized B-cell tailored dosing of ocrelizumab in patients with multiple sclerosis during the COVID-19 pandemic. Mult Scler J.

[19] Rolfes L, Pawlitzki M, Pfeuffer S (2021). Ocrelizumab extended interval dosing in multiple sclerosis in times of COVID-19. Neurol Neuroimmunol Neuroinflamm.

[20] Santagostino A, Garbaccio G, Pistorio A, Bolis V, Camisasca G, Pagliaro PGM (1999). An Italian national multicenter study for the definition of reference ranges for normal values of peripheral blood lymphocyte subsets in healthy adults. Haematologica.

[21] Kalincik T, Cutter G, Spelman T (2015). Defining reliable disability outcomes in multiple sclerosis. Brain.

[22] Cohen BA (2019). Late-onset neutropenia following ocrelizumab therapy for multiple sclerosis. Neurology.

[23] Marrodan M, Laviano J, Oneto S (2021). Rituximab- and ocrelizumab-induced early- and late-onset neutropenia in a multiple sclerosis patient. Neurol Sci.

[24] Graf J, Mares J, Barnett M (2021). Targeting B cells to modify MS, NMOSD, and MOGAD: part 1. Neurol Neuroimmunol Neuroinflamm.

[25] Baker D, Jacobs BM, Gnanapavan S (2019). Plasma cell and B cell-targeted treatments for use in advanced multiple sclerosis. Mult Scler Relat Disord.

[26] Jelcic I, Al Nimer F, Wang J (2018). Memory B cells activate brain-homing, autoreactive CD4+ T cells in multiple sclerosis. Cell.

[27] Machado-Santos J, Saji E, Tröscher AR (2018). The compartmentalized inflammatory response in the multiple sclerosis brain is composed of tissue-resident CD8+ T lymphocytes and B cells. Brain.

[28] Reali C, Magliozzi R, Roncaroli F (2020). B cell rich meningeal inflammation associates with increased spinal cord pathology in multiple sclerosis. Brain Pathol.

[29] Monaco S, Nicholas R, Reynolds R, Magliozzi R (2020). Intrathecal inflammation in progressive multiple sclerosis. Int J Mol Sci.

[30] Moccia M, Haider L, Eshaghi A (2022). B cells in the CNS at postmortem are associated with worse outcome and cell types in multiple sclerosis. Neurol Neuroimmunol Neuroinflamm.

[31] Jain RW, Yong VW (2021). B cells in central nervous system disease: diversity, locations and pathophysiology. Nat Rev Immunol.

[32] Göbel K, Ruck T, Meuth SG (2018). Cytokine signaling in multiple sclerosis: lost in translation. Mult Scler J.

[33] Choi SR, Howell OW, Carassiti D (2012). Meningeal inflammation plays a role in the pathology of primary progressive multiple sclerosis. Brain.

[34] Roodselaar J, Zhou Y, Leppert D (2021). Anti-CD20 disrupts meningeal B-cell aggregates in a model of secondary progressive multiple sclerosis. Neurol Neuroimmunol Neuroinflamm.

[35] Muñoz U, Sebal C, Escudero E (2021). Main role of antibodies in demyelination and axonal damage in multiple sclerosis. Cell Mol Neurobiol.

[36] Sádaba MC, Tzartos J, Paíno C (2012). Axonal and oligodendrocyte-localized IgM and IgG deposits in MS lesions. J Neuroimmunol.

[37] Genain CP, Cannella B, SLH & CSR (1999). Identification of autoantibodies associated with myelin damage in multiple sclerosis. Nat Med.

[38] Novi G, Ivaldi F, Sbragia E (2020). Ocrelizumab does not impair B- and T-cell responses to primary VZV infection in a patient with MS. Neurol Neuroimmunol Neuroinflamm.

[39] Fecteau JF, Roy A, Néron S (2009). Peripheral blood CD27+ IgG+ B cells rapidly proliferate and differentiate into immunoglobulin-secreting cells after exposure to low CD154 interaction. Immunology.

[40] Fransen NL, Hsiao CC, Van Der Poel M (2020). Tissue-resident memory T cells invade the brain parenchyma in multiple sclerosis white matter lesions. Brain.

[41] Brand RM, Friedrich V, Diddens J, Pfaller M (2021). Anti-CD20 depletes meningeal B cells but does not halt the formation of meningeal ectopic lymphoid tissue. Neurol Neuroimmunol Neuroinflamm.

[42] Barkhof F, Kappos L, Wolinsky JS (2019). Onset of clinical and MRI efficacy of ocrelizumab in relapsing multiple sclerosis. Neurology.

[43] Zappulo E, Buonomo AR, Saccà F (2019). Incidence and predictive risk factors of infective events in patients with multiple sclerosis treated with agents targeting CD20 and CD52 surface antigens. Open Forum Infect Dis.

[44] Moccia M, de Stefano N, Barkhof F (2017). Imaging outcome measures for progressive multiple sclerosis trials. Mult Scler.

[45] Tur C, Moccia M, Barkhof F (2018). Assessing treatment outcomes in multiple sclerosis trials and in the clinical setting. Nat Rev Neurol.

